# Full-Thickness Rotator Cuff Tears Can Be Safely Treated With a Resorbable Bioinductive Bovine Collagen Implant: One-Year Results of a Prospective, Multicenter Registry

**DOI:** 10.1016/j.asmr.2021.07.009

**Published:** 2021-08-20

**Authors:** Louis F. McIntyre, Sean McMillan, Scott W. Trenhaile, Shariff K. Bishai, Brandon D. Bushnell

**Affiliations:** a Orthopedic Partners, Nashville, Tennessee, United States; b Virtua Medical Center, Burlington, New Jersey, United States; c OrthoIllinois, Rockford, Illinois, United States; d Associated Orthopedists of Detroit, Detroit, Michigan, United States; e Department of Orthopedic Surgery, Harbin Clinic, Rome, Georgia, United States

## Abstract

**Purpose:**

The purpose of this study was to prospectively collect safety and efficacy data in a large group of patients undergoing arthroscopic repair of full-thickness rotator cuff tears augmented with a resorbable bioinductive bovine collagen implant designed to promote healing.

**Methods:**

Seventeen centers across the United States enrolled patients in an institutional review board-approved registry to collect outcomes data on the implant. Patients undergoing surgical management of full-thickness rotator cuff tears augmented with the implant were enrolled. Inclusion criteria were age of ≥21 years, willingness to participate and the ability to read and speak English. Exclusion criteria included hypersensitivity to bovine-derived products. Patients were assessed before and after surgery at up to 1 year with outcomes including the single-assessment numeric evaluation (SANE), Veterans RAND 12-Item (VR-12) mental components and physical components (VR-12 PCS), American Shoulder and Elbow Surgeons (ASES), and Western Ontario Rotator Cuff (WORC) outcome measures. Ad hoc analyses were performed to compare these outcomes at all time points depending on tear size (small/medium vs large/massive). Serious complications were collected.

**Results:**

Of 210 patients enrolled, 192 had 1-year follow-up data available. The patients experienced statistically significant improvement between baseline and 1 year for mean SANE, VR-12 PCS, ASES, and WORC scores (40.0-82.0, 33.5-47.3, 46.2-87.8, and 36.2-81.0, respectively; *P* < .001 for all results). Ad-hoc analysis demonstrated that similar results were obtained at 1 year regardless of tear size. Twenty patients (10.4%) experienced serious complications (10.4%), including revision surgery (n = 18), proximal humerus fracture/partial subscapularis tear resulting from multiple falls (n = 1), and adhesive capsulitis (n = 1).

**Conclusions:**

The safety and efficacy of a bioinductive implant in the surgical management of full-thickness rotator cuff tears at 1 year was shown in this study. Implant efficacy appears to be comparable regardless of the underlying tear size.

**Level of Evidence:**

Level IV, therapeutic case series.

Rotator cuff disease is degenerative in nature and age related. Changes in the rotator cuff tendons and footprint can lead to tensile overload, disorientation of collagen fibers, myxoid degeneration, chondroid metaplasia, and fatty infiltration.^1^^,^^2^ Thus the spectrum of rotator cuff disease ranges from simple inflammatory tendinitis to fibrosis, delamination, partial-thickness tearing, and full detachment of the rotator cuff footprint. This process can be affected by trauma, in the form of acute tears, as well as by the local individual anatomic environment or concomitant systemic disease.

Surgical rotator cuff repair has been recommended for the treatment of symptomatic tendon tears for a century.^3^ However, healing and resolution of symptoms after arthroscopic repair are thought to depend in part on underlying physiological factors contributing to the degenerative nature of these tears, such as tendon vascularity, tissue quality, and footprint pathology.^4^, ^5^, ^6^, ^7^ As a result, there is great interest in using biological treatments to augment and enhance the healing environment in rotator cuff repair.^8^, ^9^, ^10^, ^11^ The use of a resorbable bioinductive bovine collagen implant has been studied and found to be safe and effective in the surgical treatment of both partial- and full-thickness tears.^12^, ^13^, ^14^, ^15^, ^16^, ^17^

The purpose of this study was to prospectively collect safety and efficacy data in a large group of patients undergoing arthroscopic repair of full-thickness rotator cuff tears augmented with a resorbable bioinductive bovine collagen implant designed to promote healing. The study’s hypothesis was that the patient-reported outcomes (PROs) and safety profile of the implant at 1 year would be confirmed.

## Methods

An institutional review board-approved prospective data registry study was created to collect PROs using established survey instruments following treatment with a resorbable collagen implant (REGENETEN; Smith & Nephew, Andover, MA). Seventeen centers (18 surgeons) across the United States enrolled patients with the inclusion criteria of age at least 21 years, willingness to be part of the data collection effort, and the ability to read and speak English. Exclusion criteria included hypersensitivity to bovine-derived products. Institutional review board approval was obtained for each investigational site. The study was performed in compliance with the Declaration of Helsinki.

Baseline data included medical history of diabetes, smoking, worker’s compensation status, and shoulder injury. Details including timing of injury, history of trauma, duration of symptoms and previous treatments were recorded. Operative data included Cofield grade,^18^ concomitant shoulder pathology, and additional surgical procedures. PROs including American Shoulder and Elbow Surgeons (ASES), single-assessment numeric evaluations (SANE), Veterans RAND 12-Item (VR-12) mental and physical components, and Western Ontario Rotator Cuff (WORC) scores were collected before surgery and assessed for postoperative improvements at 2 weeks, 6 weeks, 12 weeks, 6 months, and 1 year, which constituted the study’s primary efficacy variable. Secondary efficacy variables were postoperative recovery parameters including time in a sling, completed number of physical therapy visits, and return to work (employed patients only), driving, and athletics (including overhead athletics); as well as the occurrence of serious complications (defined as serious adverse events, serious adverse device deficiency, and/or revision or reoperation surgery on index shoulder), which was documented throughout the course of the study by the investigators.

Patient medical history and clinical conditions were assessed with routine documentation and reviewed in the context of registry eligibility criteria. Patient surveys were administered in accordance with the registry’s study visit schedule and were completed by the patients either during their clinic visits or remotely using a unique, secure electronic case report form account access information (eClinicalOS, IBM Clinical Development, Morrisville, NC). Outcomes data were recorded and entered through an internet-based electronic data capture system. Electronic case report forms were configured to collect all outcomes data and read/write protections were established to ensure each study center could only enter and view data from their own patients.

**Figure 1:**
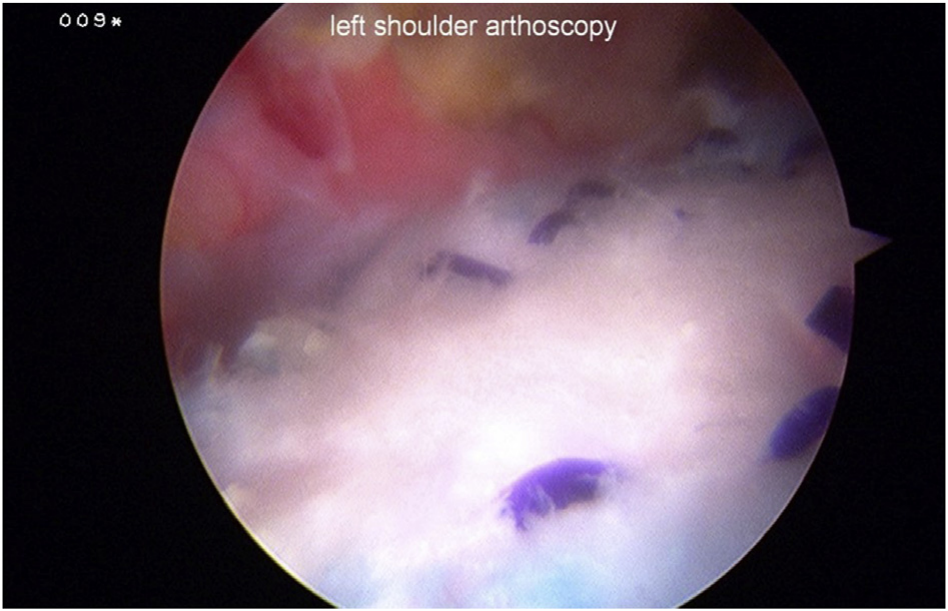
Left shoulder with bioinductive implant applied over rotator cuff repair, as viewed from posterior portal with patient in lateral decubitus position.

Patients had their tears repaired with the operating surgeon’s preferred repair techniques and implants, followed by application of a resorbable collagen implant with a technique published previously (Fig 1).^17^ The postoperative rehabilitation protocols were determined by each operating surgeons’ preferences.

The study implant consists of 3 components: a bioinductive implant made from highly purified reconstituted purified type I collagen fibers derived from bovine tendon and designed to completely resorb within 6 months; polylactic acid tendon anchors designed to functionally degrade by 6 months and completely resorb within 12 months; and polyetheretherketone bone anchors that are not resorbable.

### Statistical Analysis

The mean (± standard deviation [SD]) for each PRO was calculated at baseline and each subsequent follow up. Repeated measures analysis of variance was used to assess statistical significance. Established literature was used to determine the minimal clinically important difference for the ASES, SANE, VR-12 mental components (MCS), VR-12 physical component (PCS), and WORC scores.^19^, ^20^, ^21^ A power analysis performed on the primary shoulder-specific efficacy variables (change in all PROs over baseline), indicated power >99%. Power analysis on the VR-12 MCS as a general quality-of-life measure showed 47% power. Ad hoc analyses were performed to compare PROs at all time points depending on Cofield grade tear size (i.e., small/medium vs large/massive). Paired *t*-tests were performed to test the difference between the means at 1-year follow-up compared to the baseline measurements. Means (± SD) were reported for postoperative recovery parameters (e.g., time in a sling). Continuous variables were summarized with mean and SDs, and categorical variables were summarized with the number and percentage. Paired *t*-tests were performed to test the difference between the means of the follow-up compared to the baseline measurements. Analyses were performed with SAS version 9 (copyright 2002-2012; SAS Institute Inc., Cary, NC). Statistical significance was set at *P* < .05, and normality was tested using Shapiro-Wilks.

## Results

Between April 2016 and December 2018, there were 210 patients with full-thickness tears enrolled in the data registry (Table 1). Eighteen patients were lost to follow-up, leaving 192 (91.4%) available for analysis at 1 year (mean follow up, 378.5 days [range, 99-775]).

**Table 1:** Patient Demographics and Clinical Characteristics (n = 210)

Variable	Value
Age (yr)	
Mean ± SD	57.5 ± 8.9
Median (range)	58.0 (32-90)
Sex, N (%)	
Female	79 (37.6)
Male	131 (62.4)
Body mass index, kg/m^2^	
Mean ± SD	30.4 ± 6.2
Median (range)	29.7 (19.2-57.4)
History of symptoms (months)	
Mean ± SD	20.7 (43.4)
Median (range)	6.0 (0-360)
Time of injury, N (%)	
Acute	88 (41.9)
Acute-on-chronic	41 (19.5)
Chronic	81 (38.6)
Surgery type, N (%)	
Primary	173 (82.4)
Revision	37 (17.6)
Diabetes, N (%)	
No	171 (81.4)
Yes	39 (18.6)
Smokers, N (%)	
No	176 (83.8)
Yes	34 (16.2)
Workers’ compensation, N (%)	
No	180 (85.7)
Yes	30 (14.3)
Other musculoskeletal disorders, N (%)	
No	168 (80.0)
Yes	42 (20.0)
Chronic opioid/narcotics use, N (%)	
No	190 (90.5)
Yes	20 (9.5)

SD, standard deviation.

Intraoperative arthroscopic visualization confirmed 210 full-thickness tears, with 12 graded as small (5.7%), 92 as medium (43.8%), 75 as large (35.7%), and 31 as massive (14.8%). Concomitant surgical procedures included acromioplasty (85.7%), acromioclavicular joint resection (51.4%), capsular release (10.5%), and biceps surgery (72.8%).

**Figure 2:**
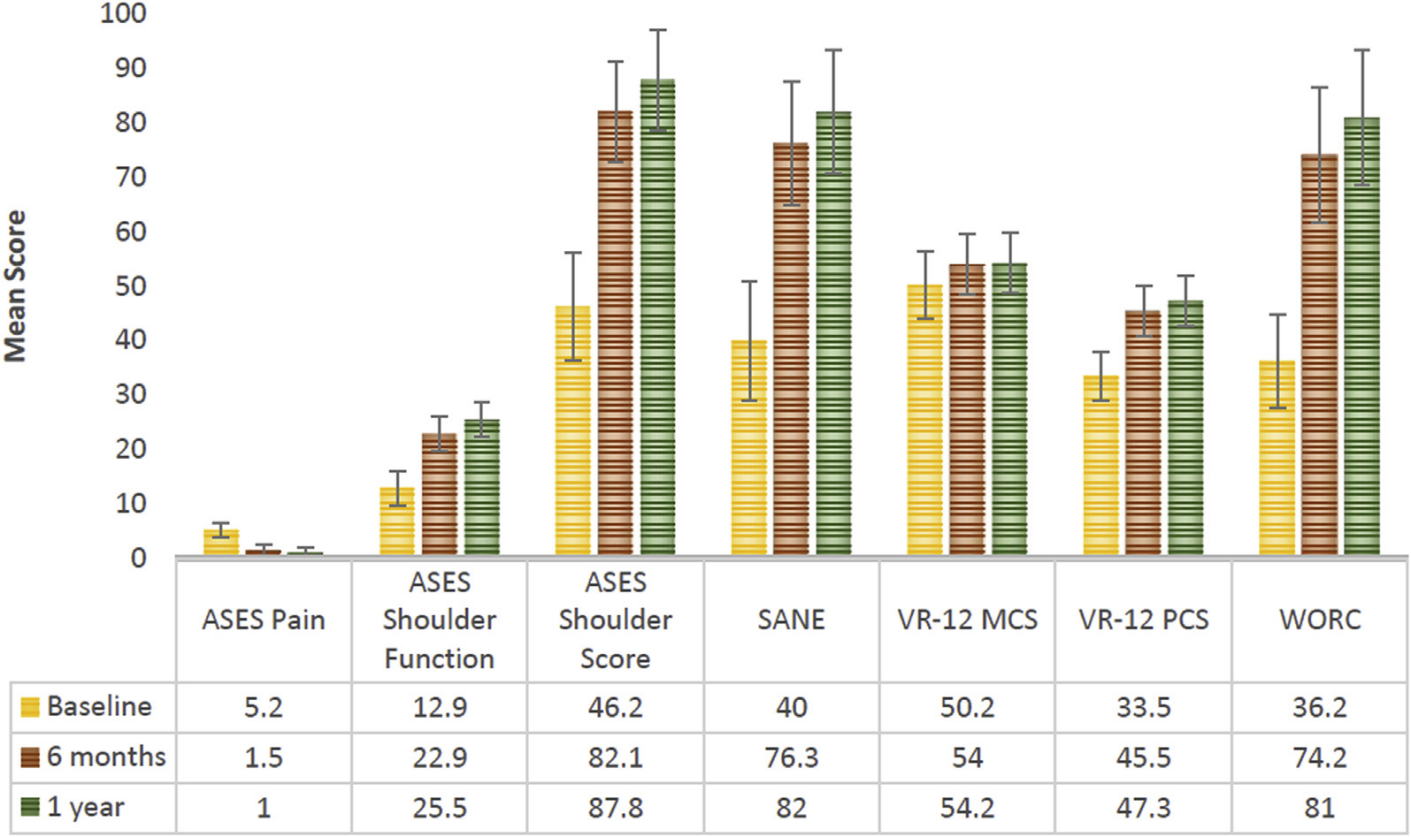
Patient-reported outcomes at baseline, 6 months, and 1 year. *P* value refers to an overtime comparison with baseline. *P* value for VR-12 MCS was .002 at 6 months and .060 at 1 year; at all other points it was <.001. ASES, American Shoulder and Elbow Surgeons; SANE, single-assessment numeric evaluation; VR-12, Veterans RAND 12-Item; VR-12 MCS, VR-12 mental component; VR-12 PCS, VR-12 physical component; WORC, Western Ontario Rotator Cuff.

Patients in the overall cohort exhibited statistically significant improvement in outcomes for the SANE, VR-12 PCS, ASES and WORC over 1 year of registry follow-up (Fig 2). The VR-12 MCS was statistically no different over the same time. The minimal clinically important difference was achieved at 1 year for SANE in 84.3% patients (161/191), for VR-12 MCS in 40.3% (77/191), for VR-12 PCS in 78.5% (150/191), for ASES in 90.5% (86/95), and for WORC in 87.2% (116/133).

An ad-hoc analysis was conducted comparing PROs at all time points between those with small/medium and large/massive tear sizes. Only mean baseline SANE scores significantly differed between the 2 groups (43.1 for small/medium vs 37.0 for large/massive; *P* = .0428). At all other time points, there was no significant difference between the groups for any of the measured PROs.

Average time in a sling for 188 patients was 36.3 days (SD, 16.8). Return to driving occurred after an average of 24.0 days (SD, 25.8) in 135 patients and work after 48.4 days (SD, 52.1) in 128 patients. Return to non-overhead athletics averaged 105.4 days (SD, 77.2) in 71 patients and overhead athletics 131.7 days (SD, 77.3) in 42 patients. Total number of physical therapy visits among 144 patients averaged 21.8 (SD, 16.2).

Twenty patients (10.4%) experienced serious complications, including 18 (9.4%) who underwent revision surgeries (Table 2). Among the 11 patients who underwent revision because of retear/failure to heal, 5 had tears categorized as medium at baseline, 3 as large, and 3 as massive. Two patients experiencing retear/failure to heal had undergone an ipsilateral rotator cuff repair before the index procedure of the study. Three patients experienced retear/failure to heal as a result of accidental trauma and 2 as a result of individual noncompliance (failure to comply with postoperative instructions).

**Table 2:** Summary of serious complications observed throughout 1-year follow up

Revisions	
Incidence of revision surgery	22
Patients undergoing revision surgery	18
Reason for revision∗	
Infection†	3
Shoulder stiffness/adhesive capsulitis	3
Clinically significant bursitis (e.g., severe pain, restricted movement, etc.)	1
Retear/failure to heal	11
Implant displacement (proud staple) after a fall	1
Time to first revision surgery, days (SD)	160.5 (98.7)
Additional serious complications	
Proximal humerus fracture/partial subscapularis tear resulting from multiple falls, and resolved with over-the-counter pain management, immobilization and physical therapy	1
Postoperative adhesive capsulitis resolved with manipulation under anesthesia	1

SD, standard deviation.

∗ Revisions can be attributed to multiple reasons.

† Includes 2 recurrent infections (1 stitch abscess that progressed to a deep infection and 1 deep infection with *Staphylococcus epidermidis*) and 1 stitch abscess that progress to an infection, all of which resolved with treatment.

## Discussion

The most important finding of the current study is that a comparable efficacy and safety profile was obtained 1 year after treatment of full-thickness rotator cuff tears augmented with the study implant, even as the cohort of patients in this registry was expanded substantially from an earlier analysis^12^ (83 to 210, respectively).

Several previous clinical studies have assessed this implant in the treatment of full-thickness rotator cuff tears. A 2015 analysis of magnetic resonance imaging in 8 patients reported increased tendon thickness at 3 months after surgery, which was sustained out to 2 years, as compared with published average normal values.^14^ Four years later, a separate publication concluded the implant led to new tendon formation and a 96% healing rate (as observed on magnetic resonance imaging and ultrasound scanning) in 23 patients.^22^ Most recently, a 2020 publication of interim results from a prospective multicenter study of 115 patients with full-thickness rotator cuff tears receiving this implant as adjunctive to single- or double-row repair reported favorable retear rates and improved clinical function at 1 year.^23^

Although these studies have advanced the understanding of this implant as an adjunctive treatment for full-thickness rotator cuff tears, the current analysis has several proposed advantages that contribute to the body of knowledge surrounding its use. It represents the largest population reported to date of full-thickness rotator cuff tears treated with this implant, with further power drawn from the high proportion of patients available at final follow-up. It used a study design intended to mirror real-world practices, with limited inclusion criteria proposed to better capture the wide breadth of patient and full-thickness tear causes encountered by clinicians. Results were obtained from numerous centers and surgeons across the United States, indicating that its positive outcomes may be generalizable. Finally, the 1-year follow-up period reflects standard of care in real-world practices and is appropriate for determining our primary variables, because evidence suggests that clinically significant improvements in PROs are not observed beyond this postoperative time period in patients undergoing rotator cuff repair.^24^

Additionally, findings from an ad-hoc analysis (small/medium vs large/massive tears) demonstrated that similar results are obtained at 1 year regardless of tear type. Because increasing full-thickness rotator cuff tear size has been correlated with worsening clinical symptoms,^25^^,^^26^ the comparable results observed in both treatment groups may be indicative of the value of augmentation with the study implant.

The manner in which this implant alters the biologic and biomechanical environment is still unclear and the subject of ongoing research. However, there is a growing body of literature documenting the use of processed collagen grafts to augment rotator cuff repair. These studies have demonstrated magnetic resonance imaging evidence of increased tendon thickness with use of the implant.^13^, ^14^, ^15^, ^16^

Results from the current analysis offer further evidence that repair surgery augmented with this bioinductive implant has efficacy across full-thickness rotator cuff tears of various causes. Existing nomenclature defines rotator cuff tears as being acute, denoting a traumatic origin, and degenerative or chronic, denoting an atraumatic etiology, with acute-on-chronic representing a combination of these 2 causes. However, there is an emerging consensus that these terms do not accurately reflect the often overlapping and complex causes of rotator cuff tears.^27^ Even tears with traumatic origins have been shown to have existing pathologic evidence of degeneration, providing the means for the tears to occur.^28^

There has also been a shift in how best to treat rotator cuff tears. Orthopaedic surgeons have approached rotator cuff tears as a mechanical problem deserving a mechanical solution for most of the last 100 years.^3^ In recent years, rotator cuff tendon disease is considered by an increasing number of researchers and surgeons as more of a degenerative phenomenon exacerbated by occupational, environmental and medical factors.^2^ Research focusing on changes in tendon vascularity, cell and collagen content, and various chemical messengers are increasing the understanding of rotator cuff tendons in both healthy and diseased states.^29^ Even the concept of a tear, which implies a traumatic origin, is giving way to recognition of a gradual degenerative and senescent process where the tendon enthesis becomes incompetent over time.^2^ This is reflected in recent changes to the nomenclature, most predominately used in Europe, with retears often being referred to as tear recurrence.

The historical mechanical focus is shifting toward a biologic one where the interplay of collagen, blood vessels, bone and chemical messengers all play a role in treating rotator cuff disease. There is still an important role for the mechanical treatment of rotator cuff disease and reattachment of the tendon back to the footprint, but there is increasing recognition that the limitations of that treatment are related to biology and not necessarily only to the biomechanical limits of repair techniques. The incorporation of biologic treatments into the mechanical reconstruction of the muscle-tendon unit may therefore provide the means for improving underlying rotator cuff pathology and increasing the success of postoperative outcomes.

Regarding the current study’s additional recovery outcomes, average time in a sling for patients (36.3 days) compares well with the literature, which indicates 4 to 6 weeks of sling time for patients treated with more traditional methods.^30^, ^31^, ^32^, ^33^ Decreased time in a sling presents the opportunity to resume activities of daily living, including driving and work, sooner. Patients treated with the implant and repair technique returned to driving an average of 24 days after operation, which compares favorably to reports in the literature of an average return of 8 weeks.^34^ Return to sport averaged 105.4 days, which compares favorably to an average return of 6.9 months in the literature.^35^

The most common reason for initial revision surgery was retear/failure to heal (11 patients; 5.7%). This occurred in 5 tears (5.4%) characterized as medium at baseline, 3 (4.0%) as large, and 3 (9.7%) as massive. This compares favorably with the United Kingdom Rotator Cuff Trial study out of the United Kingdom, the largest known prospective study of rotator cuff repairs, which reported 32%, 53%, and 73% rates of retear for medium, large, and massive full-thickness tears, respectively, at 1 year.^36^ It has been reported that retear rates for medium and large full-thickness tears level off by 10 to 15 months’ follow-up,^37^ and it is therefore probable that the current study time frame has overlapped with the main period of postoperative risk for this adverse outcome.

### Limitations

Limitations of the study include a level IV design with inherent selection bias and lack of a control population. The use of the implant was at the discretion of the practicing surgeon without evidence-based indications for use and no comparison group available for analysis. As such, no conclusions regarding the utility of the implant compared to current techniques with or without augmentation can be drawn. Future high-level comparison studies will be necessary to outline indications and clinical situations where the implant will add value to healing rates and patient outcomes. The registry design of this study was intended as a real-world evidence capture activity, reflecting the typical clinical practices of surgeons treating patients with rotator cuff tears. As such, patients were followed out to 1 year, and additional outcomes of interest (e.g., range of motion, strength, radiographic data) were not formally collected, all of which can be viewed as limitations. The results also reflect different surgeon preferences regarding rehabilitation protocols and the optimal time to return to certain activities, potentially adding unforeseen bias. Finally, the ad-hoc analysis comparing PROs based on tear size at baseline was not sufficiently powered and therefore should only be considered hypothesis-generating.

## Conclusion

The safety and efficacy of a bioinductive implant in the surgical management of full-thickness rotator cuff tears at 1 year was shown in this study. Implant efficacy appears to be comparable regardless of the underlying tear size.

## Supplementary Data

ICMJE author disclosure forms
