# Supplementary material for: Full-Thickness Rotator Cuff Tears Can Be Safely Treated With a Resorbable Bioinductive Bovine Collagen Implant: One-Year Results of a Prospective, Multicenter Registry
Source: Arthrosc Sports Med Rehabil. 2021 Aug 20;3(5):e1473–9. doi: 10.1016/j.asmr.2021.07.009 (PMC8527318; doi:10.1016/j.asmr.2021.07.009)
Supplement: Supplemental table [file mmc2.docx]

**Table 2.** Patient-reported outcomes at baseline and all follow-up points.

|  | **Baseline** | | **2 weeks** | | | **6 weeks** | | | **3 months** | | | **6 months** | | | **1 year** | | |
| --- | --- | --- | --- | --- | --- | --- | --- | --- | --- | --- | --- | --- | --- | --- | --- | --- | --- |
| **Variable** | **N** | **Mean (SD)** | **N** | **Mean (SD)** | **P-value (∆)** | **N** | **Mean (SD)** | **P-value (∆)** | **N** | **Mean (SD)** | **P-value (∆)** | **N** | **Mean (SD)** | **P-value (∆)** | **N** | **Mean (SD)** | **P-value (∆)** |
| ASES Pain | 208 | 5.2 (2.8) | 205 | 3.6 (2.7) | <.001 | 205 | 2.6 (2.4) | <.001 | 203 | 2.0 (2.3) | <.001 | 194 | 1.5 (2.1) | <.001 | 189 | 1.0 (2.0) | <.001 |
| ASES Shoulder Function | 134 | 12.9 (6.3) | 145 | 4.1 (4.8) | <.001 | 138 | 9.1 (6.2) | <.001 | 131 | 17.1 (6.9) | <.001 | 126 | 22.9 (7.1) | <.001 | 120 | 25.5 (6.2) | <.001 |
| ASES Shoulder Score | 133 | 46.2 (19.8) | 144 | 40.1 (15.4) | 0.004 | 138 | 53.8 (15.8) | 0.001 | 130 | 70.3 (18.1) | <.001 | 125 | 82.1 (18.5) | <.001 | 118 | 87.8 (18.4) | <.001 |
| SANE | 209 | 40.0 (21.8) | 205 | 22.1 (20.3) | <.001 | 204 | 37.7 (21.7) | 0.150 | 203 | 64.6 (21.3) | <.001 | 195 | 76.3 (22.5) | <.001 | 191 | 82.0 (22.8) | <.001 |
| VR-12 MCS | 150 | 50.2 (12.3) | 155 | 48.2 (12.5) | 0.033 | 144 | 50.1 (12.7) | 0.250 | 137 | 53.2 (11.9) | 0.121 | 150 | 54.0 (10.0) | 0.002 | 149 | 54.2 (11.1) | 0.060 |
| VR-12 PCS | 150 | 33.5 (9.1) | 155 | 31.5 (8.8) | 0.081 | 144 | 34.5 (8.1) | 0.275 | 137 | 40.6 (8.9) | <.001 | 150 | 45.5 (9.2) | <.001 | 149 | 47.3 (9.1) | <.001 |
| WORC | 175 | 36.2 (17.3) | 171 | 33.3 (14.6) | 0.024 | 174 | 41.0 (16.3) | <.001 | 165 | 58.7 (23.1) | <.001 | 161 | 74.2 (25.3) | <.001 | 157 | 81.0 (24.7) | <.001 |

Abbreviations: ASES = American Shoulder and Elbow Surgeons; MCS = mental component score; PCS = physical component score; SANE = single-assessment numeric evaluation; VR-12 = Veterans RAND 12-Item; WORC = Western Ontario Rotator Cuff.

Note: Patient numbers at each time point for each score differs depending on how, when, and whether or not patients filled out all the recommended data collection forms.

P value refers to an overtime comparison
